# Platinum Salts in Patients with Breast Cancer: A Focus on Predictive Factors

**DOI:** 10.3390/ijms20143390

**Published:** 2019-07-10

**Authors:** Mattia Garutti, Giacomo Pelizzari, Michele Bartoletti, Matilde Clarissa Malfatti, Lorenzo Gerratana, Gianluca Tell, Fabio Puglisi

**Affiliations:** 1 U.O.C Oncologia Medica, Fondazione Policlinico Universitario Agostino Gemelli IRCCS, 00168 Roma, Italy; 2 Department of Medicine (DAME), University of Udine, 33100 Udine, Italy; 3 Dipartimento di Oncologia Medica, Centro di Riferimento Oncologico di Aviano (CRO) IRCCS, 33081 Aviano, Italy

**Keywords:** platinum, breast cancer, *BRCA*, BRCAness, homologous recombination repair, base excision repair

## Abstract

Breast cancer (BC) is the most frequent oncologic cause of death among women and the improvement of its treatments is compelling. Platinum salts (e.g., carboplatin, cisplatin, and oxaliplatin) are old drugs still used to treat BC, especially the triple-negative subgroup. However, only a subset of patients see a concrete benefit from these drugs, raising the question of how to select them properly. Therefore, predictive biomarkers for platinum salts in BC still represent an unmet clinical need. Here, we review clinical and preclinical works in order to summarize the current evidence about predictive or putative platinum salt biomarkers in BC. The association between *BRCA1/2* gene mutations and platinum sensitivity has been largely described. However, beyond the mutations of these two genes, several other proteins belonging to the homologous recombination pathways have been linked to platinum response, defining the concept of BRCAness. Several works, here reviewed, have tried to capture BRCAness through different strategies, such as homologous recombination deficiency (HRD) score and genetic signatures. Moreover, p53 and its family members (p63 and p73) might also be used as predictors of platinum response. Finally, we describe the mounting preclinical evidence regarding base excision repair deficiency as a possible new platinum biomarker.

## 1. Introduction

Breast cancer (BC) is the most commonly diagnosed cancer in women and the first cause of cancer death in women [1]. Despite recent therapeutic advances, metastatic BC (mBC) remains a lethal disease, and there is fervent interest in the discovery of new drugs that may change the natural history of the disease [2]. Currently, BC is classified into different subtypes according to the expression of estrogen and progesterone receptors and the overexpression of HER2 protein. This classification has a practical implication, since each cancer subtype requires different medical treatments [2]. Of note, triple-negative BC (TNBC) is characterized by negative hormonal receptor status and negative HER2 status [3].

Platinum salts (e.g., carboplatin (CBDCA) and cisplatin (CDDP)) are long-standing compounds used in several cancer types [4]. Moreover, these agents are part of the therapeutic armamentarium for BC, especially TNBC [5,6,7]. Recently, the discovery that a defective DNA repair system (a quite common feature of cancer cells) can increase the efficacy of DNA damaging agents, has renewed interest in platinum salts [8]. The cytotoxicity of CDDP and other platinum agents is exerted through different cellular mechanisms [9,10,11,12]. Platinum salts enter into the cells through the High Affinity Copper Uptake Protein 1 (SLC31A1), whereas their efflux is guaranteed by both the ABCC2 and the Copper-Transporting P-type Adenosine Triphosphate (ATP7B) [13,14,15]. Interestingly, it has been hypothesized that platinum and copper could compete for their influx and efflux, causing a mutual interference of their transport [16,17]. Once inside the cells, platinum salts undergo aquation becoming highly reactive to many cellular targets, especially DNA [18]. At this level, platinum molecules are vulnerable to inactivation by antioxidant molecules like glutathione and metallothionein [19,20]. The aquated platinum salts react with DNA, generating monoadducts, inter- (ICL) and intra-DNA strand cross-links, and are able to distort the double helix of DNA causing single-strand breaks (SSBs) and double-strand breaks (DSBs) [11]. The principal effect of this structural distortion is the blockage of both DNA replication and DNA transcription, that if permanent, causes severe cell cycle arrest and induces cell apoptosis or necrosis [10]. In particular, triggering cell death is concentration dependent. Indeed, high CDDP doses cause necrosis, whereas chronic low doses induce apoptosis [21]. Notably, Becker et al. [22] recently demonstrated that CDDP and other platinum-containing regimens were also able to directly interfere with mRNA translation, interacting with the nascent transcript and generating similar RNA adducts. Generally, DNA bulky lesions generated by platinum drugs are efficiently repaired by nucleotide excision repair and homologous recombination (HR) pathways [18]. Moreover, several studies have demonstrated that base excision repair (BER) also plays a role in mediating cisplatin cytotoxicity [23,24,25,26,27,28,29,30,31,32]. Though not being directly active on bulky DNA lesions, different studies have highlighted a role for BER in repairing the platinum-induced DNA ICLs and in modulating other indirect effects generated by the CDDP exposure.

It is, therefore, clear that a defect in one of these DNA repair pathways, particularly HR and BER, could represent a useful predictive biomarker for platinum salt sensitivity. Patient selection is crucial when using platinum salts, and the identification of predictive biomarkers still represents an unmet clinical need. Therefore, the main purpose of this review is to summarize the currently available evidence about predictive biomarkers of platinum salt sensitivity in BC, in order to integrate them into future clinical trials, and hopefully, in clinical practice.

This review will address both translational data regarding HR deficiency (HRD) as a marker of platinum salt sensitivity and new emerging preclinical biomarkers of platinum salt sensitivity.

## 2. HRD as a Biomarker of Platinum Sensitivity

### 2.1. The HR Pathway

Once a DSB occurs, cells activate two different DNA repair mechanisms, depending on the phase of the cell cycle [33]: the HR system or the non-homologous end joining (NHEJ) system. Homologous recombination, which is mostly active during S- and G2-phases of the cell cycle [34], repairs the DSBs by using a homologous DNA molecule, therefore acting as an error-free repair mechanism [35]. In contrast, NHEJ, which is active through the whole cell cycle [34], ligates the ends of a DSB in an error-prone way, increasing DNA mutagenicity and genomic instability [36]. 

**Figure 1:**
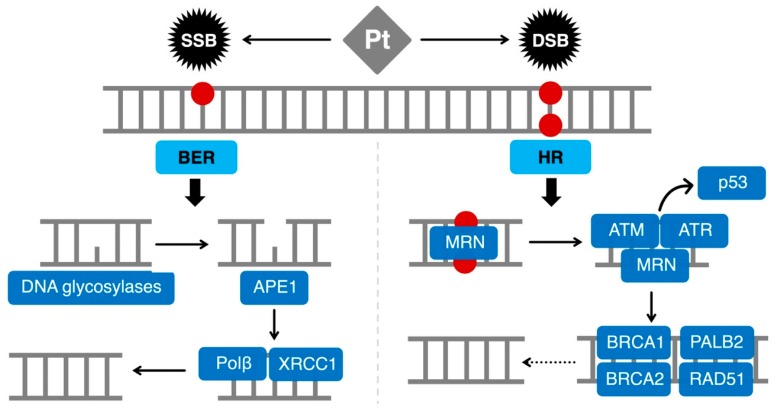
Representation of the main DNA repair pathways involved in platinum salts-induced DNA damage. SSBs are mainly repaired by the BER pathway, which needs proficient DNA glycosylases that recognize and cleave the damaged base. Then, APE1 removes the abasic site that can be sealed by Polβ and ligases. In the case of DSBs, HR plays a crucial role. The MRN complex recognizes the DSB and recruits ATM and ATR, which can eventually induce cell cycle arrest through p53. Subsequently, ATM can cause the recruitment of BRCA1, BRCA2, and PALB2 which determine the RAD51 loading and the subsequent DNA synthesis. BER: base excision repair; DBS: double-strand break; HR: homologous recombination repair; Pt: platinum salts; SSB: single-strand break.

The HR pathway consists of a high number of proteins (Figure 1) [35,37,38,39]. In a simplified view of the process, the first step involves the MRN complex (MRE11-RAD50-NBS1) that detects the DSB and recruits ATM and ATR which, in turn, activate the cell cycle checkpoints and induce cell cycle arrest through p53 [40]. Subsequently, ATM phosphorylates histone H2AX causing the recruitment of 53BP1 and the Breast Cancer Type 1 Susceptibility Protein (BRCA1) to the damaged area. At the beginning of DSB repair, BRCA1 has a crucial role. Indeed, BRCA1 controls the DSB resection and helps the transition from DSB resection to PALB2/Breast Cancer Type 2 Susceptibility Protein (BRCA2)-mediated RAD51 loading [41]. The protein RAD51 eventually forms a filament of nucleic acid and proteins allowing the alignment of broken DNA with the normal one and the subsequent synthesis of new DNA to fill the genomic gap [42].

In the case of HR deficiency, the error-prone NHEJ system becomes the central axis of DSB repair, creating genomic instability and virtual cell death [33]. As platinum salts induce DSBs, cells would need a proficient HR to survive. Therefore, cancer cells bearing an HR deficiency might be sensitive to platinum salts.

### 2.2. BRCA1/2 Mutations

The BRCA1/2 proteins represent an essential component of HR and germline mutations of their genes are detected in about 5% of unselected BC and in up to 10–20% of TNBC [43,44]. The BRCA1/2 proteins, along with others (e.g., PALB2, RAD51, and CHEK2), play a critical role in ensuring genomic stability and efficient DNA repair through the HR machinery [45,46]. On these bases, platinum compounds have been tested as a therapeutic option for BRCA-mutated tumors and TNBC, leading to promising results observed among *BRCA1* mutation carriers, treated with CDDP, in both neoadjuvant and metastatic setting [47,48] (Table 1). 

The first randomized phase III study, investigating the role of platinum salts in unselected TNBC was the Triple-Negative Breast Cancer Trial (TNT) [49]. Patients with metastatic TNBC or carrying a *BRCA* mutation were equally randomized to receive six to eight cycles of first-line carboplatin or docetaxel. In the overall population, no difference was observed among the two arms in terms of objective response rate (ORR) (ORR: 31.4% for carboplatin versus 34.0% for docetaxel; absolute difference −2.6%, 95% confidence interval (CI) −12.1 to 6.9; *p* = 0.66). Nevertheless, when looking at the pre-specified subgroup analysis according to *BRCA* mutational status, the use of carboplatin in the BRCA-mutated cohort led to a doubling in ORR compared to docetaxel (ORR: 68% for carboplatin versus 33.3% for docetaxel; absolute difference 34.7%; *p* = 0.03), with a significant heterogeneity of treatment effect for BRCA-mutated patients (interaction test: *p* = 0.01). Furthermore, longer progression-free survival (PFS) was detected for BRCA-mutated patients (median PFS: 6.8 months versus 4.4 months; interaction *p* = 0.002), even if no advantage was observed in terms of overall survival (OS) [49]. Consistently, in the phase II, non-randomized TBCRC009 trial [50], among 86 metastatic TNBC patients treated with cisplatin or carboplatin as first- or second-line therapy, BRCA-mutated patients showed higher ORR compared to those without BRCA mutations (ORR: 54.5% versus 19.7%; *p* = 0.022). Taken together, these findings confirm the biological heterogeneity of TNBC and its differential sensitivity to platinum salts, that seems to be much greater for BRCA-mutated patients. A further contribution came from the phase 2 study BROCADE, in which 290 patients, having metastatic breast cancer carrying a *BRCA1/2* mutation were randomized to receive the combination of veliparib, a poly(ADP-ribose) polymerase (PARP) inhibitor, with carboplatin plus paclitaxel versus carboplatin plus paclitaxel and versus temozolomide plus veliparib [51]. Although no difference was found in terms of PFS and OS with the addition of veliparib to carboplatin plus paclitaxel, it is noteworthy to consider the clinically relevant performance of the carboplatin plus paclitaxel arm (PFS: 12.3 months; OS: 25.9 months), that may represent a reasonable treatment for these patients, as it will be further evaluated in the phase III BROCADE 3 trial (NCT02163694).

Dealing with the neoadjuvant setting, the role of platinum salts is still controversial. A recent meta-analysis, considering data from nine randomized clinical trials, confirmed an absolute 15% increased pathologic complete response (pCR) rate, when adopting platinum-based regimens in TNBC (pCR rate from 37.0% to 52.1%; OR 1.96, 95% CI 1.46–2.62; *p* < 0.0001), even though high heterogeneity was detected among the included studies. Nevertheless, no statistical benefit was observed on both event-free survival (EFS) and OS within the same pooled analysis [6]. This discrepancy represents one of the most-debated arguments when discussing the implementation of platinum salts in the neoadjuvant treatment of TNBC, together with toxicity issues and a missing standard combination regimen. However, it is important to consider that none of these studies were designed to detect any impact on long-term outcomes. Hence, the power of the studies may not be adequate for these speculations. The two most representative randomized phase II studies conducted in this setting are the GeparSixto and the CALGB 40603 trials [52,53,54]. Both studies confirmed a pCR benefit with the addition of platinum agents to a neoadjuvant chemotherapy with taxanes and anthracyclines [52,53,54]. Nevertheless, the GeparSixto is the only study detecting a significant increase in disease-free survival (DFS) in this setting (DFS at 3 years: 86.1% versus 75%; HR: 0.56, 95% CI 0.34–0.93; *p* = 0.0244) [53], while no significant EFS difference was observed in the CALGB 40603 trial (EFS at 3 years: 76.5% versus 71.6%; HR: 0.84, 95% CI 0.58–1.22, *p* = 0.36) [55]. When looking at the BRCA-mutated subgroup of the GeparSixto trial (representing only the 17.4% of the overall population), high pCR rates were observed irrespectively of carboplatin use (pCR rates: 66.7% for non-carboplatin arm versus 65.4% for carboplatin arm; HR: 0.94, 95% CI 0.29–3.05; *p* = 0.92), confirming high chemo-sensitivity of *BRCA1/2* carriers. However, no additive effect was observed for carboplatin (interaction test: *p* = 0.58) in this subgroup [56]. Surprisingly, the *BRCA* wild-type cohort benefited the most from the addition of carboplatin, in terms of pCR (pCR rates: 36.4% for non-carboplatin arm versus 55% for carboplatin arm; HR: 2.14, 95% CI 1.28–3.58; *p* = 0.004) [55]. This observation may be justified by the presence of BRCA-like phenotypes among sporadic TNBC, but also by the use of an intensified chemotherapy regimen. Additionally, the BrighTNess trial, a randomized clinical trial evaluating the role of neoadjuvant carboplatin alone or in combination with veliparib for TNBC patients, reported similar pCR data according to *BRCA*-mutational status [57]. However, these post-hoc subgroup analyses were performed among a very limited number of *BRCA*-mutated patients (50 patients in the GeparSixto trial and 46 patients in the BrighTNess study), and their relevance remains exploratory. 

In conclusion, the neoadjuvant management of *BRCA*-mutated patients is still a matter of debate, with no definitive data supporting the utility or futility of platinum salts in this setting. Interestingly, a significant contribution will be provided by the ongoing INFORM study (NCT01670500), a randomized, phase II trial comparing four cycles of neoadjuvant cisplatin versus four cycles of doxorubicin plus cyclophosphamide among BRCA-mutated patients with early breast cancer.

### 2.3. BRCAness

Although *BRCA1* and *BRCA2* gene products are considered the major players in the HR system, cancers harboring a pathological variant of *BRCA1/2* genes represent only the tip of the iceberg of the overall BC characterized by a homologous recombination deficiency, due to the emerging subgroup of tumors that share clinico–biological features of BRCA-mutant tumors in the absence of a *BRCA1* or *BRCA2* mutation, a condition known as BRCAness [63]. Since therapies, such as platinum-based chemotherapy and PARP inhibitors, have revealed their efficacy in *BRCA1/2* mutation carriers [64,65], searching for BRCAness has become more appealing especially for those tumors characterized by poor outcomes and treatment options (Table 1). In TNBC, for example, BRCAness is found in more than 25% of cases [66]. Unfortunately, an unequivocal BRCAness biomarker is currently lacking, due to the wide spectrum of genetic and epigenetic alterations that may be involved. Among them, the most frequent genetic alteration involve *ATM*, *ATR*, *PALB2*, *CHEK1*, *CHEK2*, *RAD51*, Nijmegen breakage syndrome protein 1 (*NBS1*) and the Fanconi anaemia complementation group (*FANC*) family [63]. In addition, the modification of the cellular transcriptional activity, like those induced by *BRCA1* promoter hypermethylation, can be a cause of epigenetic BRCAness. Given the evidence that cancer with an HRD system shows a typical mutational signature produced by the error-prone NHEJ activity in repairing DSBs [67], two commercial assays detecting the main three genomic structural rearrangements founded in HRD tumors has been developed. In “myChoice HRD” (Myriad genetics) test, loss of heterozygosity (LOH), telomeric allelic imbalance (TAI), and large-scale transition (LTS) are measured and can be combined in an HRD score. The predictive power of a specific HRD threshold derived from the combined HRD score has been evaluated retrospectively by Telli et al. [58]. In this study, HR deficiency (defined as an HRD score ≥ 42 and/or the presence of *BRCA1* or *BRCA2* mutation) was evaluated as a predictor of response to neoadjuvant platinum-based chemotherapy for TNBC in two different clinical cohorts. The dichotomized HRD score was significantly associated with both RCB 0/I (no residual cancer burden or minimal residual disease) and pCR in both cohorts. Moreover, HRD was still able to significantly predict RCB 0/I and pCR also when adjusted for clinical variables [58]. Concordant results have been found in a small trial addressed to patients with early stage TNBC enrolled to receive neoadjuvant platinum-based therapy. In the exploratory analysis of this trial, HRD status and the HRD score could predict pCR (HRD status *p* = 0.0012; HRD score *p* = 0.0024) [59]. Additionally, a post-hoc analysis of the GeparSixto study tried to explore the role of HRD score in predicting pCR to neoadjuvant chemotherapy in 193 patients treated for TNBC. Homologous recombination deficiency was defined as either a high HRD score (≥42) or a *BRCA* mutation in the primary tumor. Tumors with HRD were more likely to achieve pCR than HR proficient ones (55.9% versus 29.8%, *p* = 0.001). Moreover, patients with HRD tumors showed higher pCR rates with the addition of carboplatin to the chemotherapy backbone (64.9% versus 45.2%; *p* = 0.025) [68]. In a retrospective analysis of 425 patients with TNBC treated with adjuvant doxorubicin and cyclophosphamide in the SWOG9313 trial, a high HRD score (≥42) was observed in more than a half of *BRCA* wild-type patients and it was independently associated with better DFS when adjusted for treatment and nodal status (HR 0.64, 95% CI 0.43–0.94; *p* = 0.023) as well as OS (HR 0.65, 95% CI 0.47–1.53; *p* = 0.59). Moreover, *BRCA1* promoter methylation was associated with higher HRD scores but no predictive value was established [60]. Conversely, in the previously discussed TNT trial, HRD score and *BRCA1* promoter methylation were not associated to a better response to first-line platinum salts [49]. However, since HR biomarkers in TNT were evaluated on archival tissue, a possible explanation of these findings is that the “soft BRCAness” of HRD tumors is easily revertible under the selective pressure of neo/adjuvant DNA-damaging therapy. Therefore, when tumors relapse, cancer cells may be no longer methylated and sensitive to platinum agents [49,63].

Moreover, BRCAness has not only been investigated as a predictive biomarker, but also as a new therapeutic strategy through its pharmacological induction. Intriguing results suggest that the induction of a BRCA-mutant-like phenotype could be achieved through the epigenetic silencing of *BRCA1*, enhancing platinum salts’ activity and enabling the use of targeted drugs such as PARP inhibitors [69,70].

## 3. New Emerging Biomarkers

### 3.1. Gene Signatures

Lehmann et al. [61] showed that TNBCs can be further sub-classified into six different molecular entities. Among these, basal-like 1 (BL1) and basal-like 2 (BL2) subtypes had a peculiar sensitivity to platinum salts, irrespectively to *BRCA1* mutation status, suggesting the presence of other defects in the HR pathway [61]. Concordant data came from a recent study on 465 Chinese TNBCs [71]. In that study, the authors classified TNBCs into four main classes through a multi-omics approach. Among these classes, the basal-like subtype was characterized by an HR defect, and thus, may be especially sensitive to DNA damaging agents like platinum salts. 

Another genomic classification is PAM50, which differentiates at least five BC entities [72]. Among these, the basal-like can be ideally assimilated to TNBC. However, it is important to note that not all TNBCs display a basal phenotype [73,74,75,76] and basal-like cancers account for 60–90% of TNBCs [77,78]. The basal-like molecular subtype can be approximated to the core-basal breast cancer, which is only defined by the absence of ER, PgR, HER2, and the presence of EGFR and cytokeratin 5/6 [79]. In the TNT trial reported above [49], the basal-like molecular subtype and the core-basal type were investigated as possible predictive biomarkers of CBDCA sensitivity. Interestingly, in both of these types, carboplatin performs as well as docetaxel in terms of ORR and PFS. On the contrary, in non-basal-like BCs, docetaxel outperforms carboplatin in terms of ORR and PFS. A trend toward a better ORR and PFS for docetaxel was seen in non-core-basal BCs.

### 3.2. p53 Family

Other putative biomarkers of platinum sensitivity may be represented by the *p53* status and the levels of p53 family members (p63 and p73). The protein *p53* represents a crucial node in the DNA repair pathway [80], and its defects could be causative of platinum salt cytotoxicity [81,82]. Until now, only *p53*-nonsense or frameshift mutations have been associated to response to cisplatin in TNBCs [62]. Moreover, a high incidence of *p53*-truncating mutations in *BRCA1*-mutated BCs has been observed [83]. Therefore, *p53*-truncating mutations may represent an alternative marker of *BRCA1* deficiency. 

Both p63 and p73 proteins belong to the p53 family and govern a variety of cellular functions [84]. From a molecular point of view, the p63 isoform ∆Np63 antagonizes the pro-apoptotic activity of the p73 isoform TAp73 through direct physical sequestration [85]. Cisplatin showed to promote ∆Np63 dissociation from TAp73 in vitro [86]. Free TAp73 can, therefore, trigger the pro-apoptotic cascade. In light of this evidence, it has been hypothesized that ∆Np63/TAp73 levels might represent a proxy for cisplatin sensitivity. Indeed, some data suggest that a ∆Np63/TAp73 ratio > 2 may predict a favorable outcome for cisplatin [62], though the predictive role of the ∆Np63/TAp73 ratio needs further confirmation [50].

### 3.3. BER

Generally, BER is involved in processing non-bulky DNA lesions, usually induced by oxidative, alkylating, and methylation agents [87,88,89]. Although BER is composed of few sequential steps acting to guarantee the correct DNA repair, it involves a great number of non-classical DNA-repair proteins and post-translational modifications that are finely regulated [90,91,92,93]. Among BER modulators, nucleophosmin (NPM1), p53, and XRCC1 are the most effective [88,94,95], mostly in CDDP response. 

The first BER step involves lesion-specific DNA glycosylases, including 8-oxoguanine DNA glycosylase (OGG1) [96] and uracil-DNA-glycosylase (UNG) [97], that recognize and cleave the damaged base or Uracil, thus generating an abasic site (AP) (Figure 1). Then, a specific apurinic/apyrimidinic endonuclease, called APE1, cleaves the abasic site [98,99]. Finally, the SSB generated by APE1 is sealed by other BER factors, including Polymerase β (Polβ) and ligases [100,101]. Although all the enzymes are needed for the good success of the entire BER, APE1 represents the core of the pathway, being able to coordinate every step. 

As previously proposed [23,32], the clear documentation of a novel role of BER in mediating cisplatin cytotoxicity was produced by Kothandapani et al. [26]. The authors have finely demonstrated how *Polβ*- and *UNG*-deficient MDA-MB-231 TNBC cells are more resistant to CDDP treatment. The same effect was obtained by treating cells with Methoxyamine, an inhibitor of the endonuclease activity of APE1. Although the authors specified that BER enzymes are not directly involved in the removal of cisplatin-produced ICLs on DNA, they demonstrated that BER is active in damages indirectly induced by CDDP. 

A part of CDDP cytotoxicity requires the generation of reactive oxygen species (ROS) [102]. Reactive oxygen species are mainly generated as a by-product of the aerobic mitochondrial respiration or by following continuous exposition of chemical agents [103]. When ROS levels increase, thus destabilizing the redox homeostasis of the cell, DNA, proteins, and lipids can be severely damaged. Reactive oxygen species-induced DNA damages, including the oxidation of the guanine (8oxoG), are efficiently repaired by the BER pathway. Notably, the most important side effect characterizing the use of CDDP, and to a lesser extent, of other platinum drugs, is the development of a severe peripheral neuropathy. In 2009, Preston et al. [29] demonstrated that both cisplatin and oxaliplatin increase ROS and 8oxoG levels, and then later, Kelley et al. [24] supported the hypothesis that this phenomenon might be considered a secondary effect of DNA damage induced by platinum drugs in sensory neurons. Remarkably, Kelley et al.’s [24] work has demonstrated that neuron cells, silenced for *APE1* expression, are more sensitized to CDDP and oxaliplatin, but not carboplatin, observing a decrease of cell viability and a parallel increase of apoptosis. The cause of this high mortality is associated with an increased production of ROS levels within the cells that, by destabilizing the redox cell balance, induces an increase amount of 8oxoG. On the contrary, the stimulation of the APE1 endonuclease activity significantly decreases the toxicity of CDDP on neuronal cells [104]. Moreover, the treatment with the APE1-targeted first-generation (E3330) and the novel second-generation (APX2009) agonists protect against neurotoxicity induced by platinum compounds [105,106]. This should explain the role of APE1 in mediating inflammation induced by platinum drugs, principally through its redox activity [107].

Concordantly, by applying a CRISPR-based genetics screening, it has been shown how both BER-factors XRCC1 and OGG1 are involved in restoring ICLs and ROS-mediated oxidative damages induced by platinum drugs [31], highlighting again the role of BER enzymes in protecting against platinum-induced cytotoxicity.

Taken together, these observations explain how CDDP and platinum drugs may induce DNA damage by formation of bulky DNA lesions that are efficiently repaired by the cooperation of several DNA repair pathways, including BER. At the same time, the observed increase in ROS levels and the increased sensitization effect in BER-deficient cells could explain the primary involvement of the BER pathway in response to platinum drug cytotoxicity. These important observations led to consider BER enzymes as new anti-tumoral targets to be used in combination treatments with platinum drugs. The protein APE1 can be considered a new promising target for the combination of CDDP-based chemotherapy as proposed by Wang et al. [108] in NCSLC patients. Moreover, APE1 protein expression quantification revealed higher levels in CDDP-resistant patients and was correlated with a lower OS and event-free interval (EFI). Recently, we have improved the knowledge of the role of APE1 and NPM1 in mediating the response of CDDP and carboplatin in TNBC cells lines, differing for APE1 and NPM1 protein expression [109]. As already demonstrated by Poletto et al. [28], for other cell models, CDDP induced a shuttling of APE1 and NPM1 from nucleolus to nucleus compartments, which is possibly regulated to the non-DNA repair functions of APE1 on RNA metabolism. Particularly, TNBC cells, characterized by lower levels of both APE1 and NPM1 proteins, were much more sensitized to the combined treatment of platinum drugs and inhibitors of APE1 endonuclease activity as well as inhibitors of APE1–NPM1 interaction.

## 4. Conclusions

Despite being widely used for the treatment of multiple cancer types, platinum salts are only recently gaining momentum for the treatment of breast cancer, especially TNBC. Due to the great molecular heterogeneity of this subtype, several efforts have been made to identify predictive factors capable of effectively guiding patients’ stratification, but to date, the most solid one is the *BRCA1/2* mutational status. Alternative biomarkers, such as BRCAness and BER enzymes are currently under investigation to further develop the role of platinum salts in breast cancer, not just for treatment benefit prediction but also as targets for new therapeutic strategies aimed at synergizing with this class of compounds. 

## Figures and Tables

**Table 1:** Predictive biomarkers of platinum salts efficacy in BC.

Reference	Setting	Biomarker	Treatments	Outcomes/Observations
***BRCA1/2* Mutations**				
TNT [49] (phase III)	Stage IV TNBC	*BRCA1/2*m	CBDCA versus Docetaxel	Increased ORR and PFS with Carboplatin versus Docetaxel
TBCRC009 [50] (phase II)	Stage IV TNBC	BRCA1/2m	CBDCA or CDDP	Increased ORR in BRCA1/2m versus *BRCA1/2* wt
BROCADE [51] (phase II)	Stage IV BC with BRCA1/2m	BRCA1/2m	CP versus CPV versus TV	Increased ORR and PFS with CP and CPV versus TV
GeparSixto [53] (phase II)	Stage II–III TNBC	BRCA1/2m	P+A+Bev ± CBDCA	No additive effect on pCR for carboplatin
BrighTNess [57] (phase III)	Stage II–III TNBC	BRCA1/2m	CP ± V then AC	No additive effect on pCR for carboplatin
**BRCAness**				
Telli et al. [58] (pooled analysis)	Localized TNBC	HRD score > 41	Various platinum containing regimens	Increased pCR in HRD versus HRD < 41
Kaklamani et al. [59] (phase II)	Stage I–III TNBC	HR status (HRD score + BRCA1/2m)	CBDCA + E	HRD status and the HRD score predict pCR
GeparSixto [53] (phase II)	Stage II–III TNBC	HRD status *	P+A+Bev ± CBDCA	HRD positive status associated with increased pCR versus HRD negative Adding carboplatin increased pCR in HRD positive but not in HRD negative tumors
SWOG9313 [60] (phase III)	Stage I–II TNBC	HRD status *	Concomitant versus sequential AC	HRD positive status associated with DFS. No significative trend observed with OS
**Gene Signatures and p53 Family**				
Lehmann et al. [61] (in vitro analysis)	TNBC cell lines	BL	Platinum salts	Increased sensitivity
TNT [49] (phase III)	Stage IV TNBC	BL, core-basal	CBDCA versus Docetaxel	Reduced ORR and PFS in not-BL and not-core basal
Silver et al. [62]	Stage II–III TNBC	p53 NSM	Cisplatin	Increased pCR versus not p53 NSM
Silver et al. [62]	Stage II–III TNBC	∆Np63/TAp73 ratio > 2	Cisplatin	Numerical increased pCR vs. ∆Np63/TAp73 ratio < 2
**BER**				
Kothandapani et al. [26] (in vitro analysis)	MDA-MB-231 TNBC cell line	PolB	CDDP	Upon KO, cells are resistant to CDDP treatment
Kothandapani et al. [26] (in vitro analysis)	MDA-MB-231 TNBC cell line	UNG	CDDP	Upon KO, cells are resistant to CDDP treatment
Kothandapani et al. [26] (in vitro analysis)	MDA-MB-231 TNBC cell line	APE1	CDDP	Upon MX, APE1 inhibitor, cells are resistant to CDDP treatment

A: non-pegylated liposomal doxorubicin; AC: doxorubicin + cyclophosphamide; APE1: apurinic/apyrimidinic endonuclease 1; BC: breast cancer; Bev: bevacizumab; BL: basal-like gene signature; BRCA1/2m: *BRCA1/2* mutation; CBDCA: carboplatin; CDDP: cisplatin; CP: carboplatin + paclitaxel; CPV: carboplatin + paclitaxel + veliparib; DOR: duration of the response; E: eribuline; HRD: homologous recombination deficiency; KO: knock-out; MX: Methoxyamine; NSM: non-sense or frameshift mutation; ORR: objective response rate; P: paclitaxel; PFS: progression-free survival; PolB polymerase beta; TNBC: triple-negative breast cancer; TV: temozolomide + veliparib; V: veliparib; wt: wild type. * HRD status was defined positive as either a deleterious tumor *BRCA1/2* (tBRCA) mutation or a pre-defined HRD score ≥ 42. HRD status was defined negative as either an absence of deleterious tumor *BRCA1/2* (tBRCA) mutation or a pre-defined HRD score < 42.

## Abbreviations

BC	Breast cancer
mBC	Metastatic breast cancer
TNBC	Triple-negative breast cancer
CBDCA	Carboplatin
CDDP	Cisplatin
SLC31A1	High Affinity Copper Uptake Protein 1
ATP7B	Copper-Transporting P-type Adenosine Triphosphate
ICL	Inter-DNA cross-link
SSB	Single-strand break
DSB	Double-strand break
HR	Homologous recombination repair
BER	Base excision repair
HRD	Homologous recombination deficiency
NHEJ	Non-homologous end joining
BRCA1	Breast Cancer Type 1 Susceptibility Protein
BRCA2	Breast Cancer Type 2 Susceptibility Protein
ORR	Objective response rate
OS	Overall survival
PFS	Progression-free survival
PARP	Poly(ADP-ribose) polymerase
pCR	Pathologic complete response
EFS	Event-free survival
DFS	Disease-free survival
RCB	Residual cancer burden
NPM1	Nucleophosmin
OGG1	8-Oxoguanine DNA glycosylase
UNG	Uracil-DNA-glyucosylase
Polβ	Polymerase β
ROS	Reactive oxygen species
8oxoG	Oxidized guanine
EFI	Event-free survival
